# SHM System and a FEM Model-Based Force Analysis Assessment in Stay Cables

**DOI:** 10.3390/s21061927

**Published:** 2021-03-10

**Authors:** Jan Biliszczuk, Paweł Hawryszków, Marco Teichgraeber

**Affiliations:** Faculty of Civil Engineering, Wrocław University of Science and Technology, Wybrzeże Wyspiańskiego 27, 50-370 Wrocław, Poland; jan.biliszczuk@pwr.edu.pl

**Keywords:** bridges, stay cables, durability, maintenance, monitoring, SHM, FEM

## Abstract

The Rędziński Bridge in Wrocław is the biggest Polish concrete cable-stayed bridge. It is equipped with a large structural health monitoring (SHM) system which has been collecting the measured data since the bridge opening in the year 2011. This paper presents a comparison between the measured data and the finite element method (FEM) calculations, while taking into account 7 years of data collection and analyses. The first part of this paper concerns the SHM application. In the next part, which contains comparisons between forces in cables and temperature changes throughout the structure, the measured data are presented. The third part includes SHM-based calculations and simulations with a complex FEM model to check the measured data and to estimate future measurements. The last part contains a durability assessment calculation for the cable stays.

## 1. Introduction

The structural health monitoring system (SHM) is a relatively new tool for examining the technical condition of structures. A wide description of the continuous observation methods of structures in recent years can be found, e.g., in [1]. The development over the past decades has been focused mainly on fulfilling the expectations of the maintenance administration to constantly control the structures’ behaviour. Such systems provide the user with a range of information that is only compared with the design values. However, even the most complex systems can still provide incorrect or missing information. For this purpose, the collected data should be properly verified and checked, for example, with the use of adequate numerical models of the monitored structure. In this paper, such a solution is presented for the longest concrete cable-stayed bridge in Poland, the Rędziński Bridge in Wrocław. Here, as in other cable-stayed bridges, the most important load-bearing element are the stays and their tensioning force as the effect of all loads acting on the whole structure. A simple tension force analysis from the SHM system does not deliver the possibility to determine which load (vehicle movement, temperature changes, wind impact, etc.) affects its value or fluctuates the most. For fatigue or reliability calculations, a detailed model of each stay cable should be created. Advanced studies on the work of anchorages and mutual friction of the wires in the strand can be found in [2,3,4,5]. However, they are based on purely laboratory experiments.

Most of the studies related to SHM, which were cited in this work, present the general methods of these system operations. The aim of the authors was to show how to use the collected data for scientific purposes, which is also an extension of their own research presented in [6], where the SHM system of the Rędziński Bridge and measured quantities were described, but without statistical analysis. The idea of the separation of individual load effects is presented based on data from the SHM monitoring system and FEM models of the structure. The methods of validation of these data and the possibilities of their further statistical analysis are also shown. An example of their use for fatigue calculations has been proposed, as well. The tool consisting of the big SHM system and the FEM models shows how the collected data can be used for simulations of future results. Moreover, the analysis performed in this way can provide valuable information on the design of further monitoring systems for cable-stayed bridges.

## 2. The Rędziński Bridge and its SHM System

**Figure 1:**
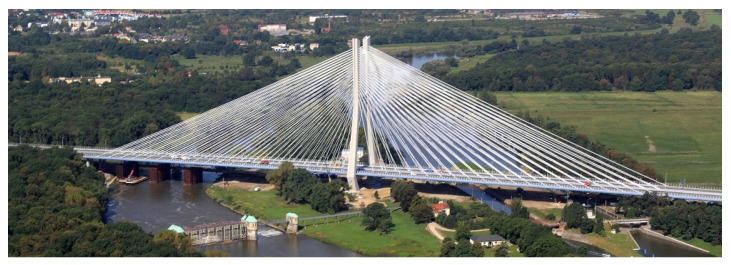
Aerial view of the Rędziński Bridge (photo: Władysław Kluczewski).

The Rędziński Bridge was built in 2011 and it is the main bridge within the Wrocław motorway ring-road. The structure is a concrete cable-stayed bridge with the spans of 49.00 m + 256.00 m + 256.00 m + 49.00 m. The H-shaped pylon is 122.00 m high (Figure 1) measured from the foundation top surface.

**Figure 2:**
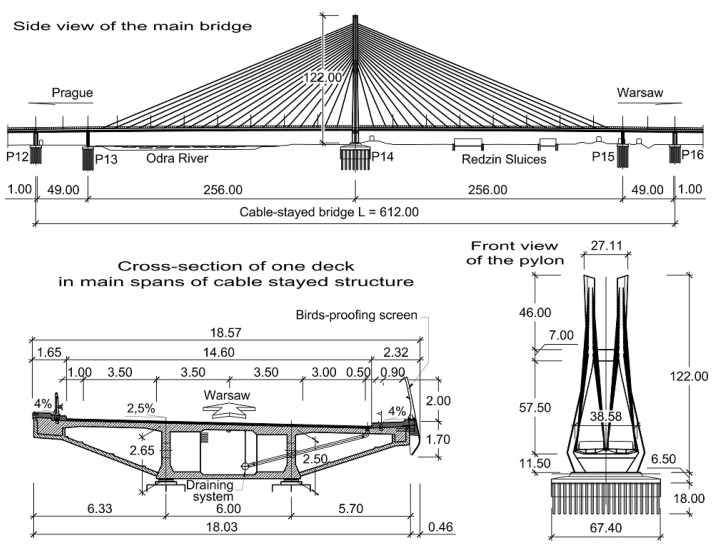
Main properties of the Rędziński Bridge [7].

The characteristic feature of the bridge is two separate box girder concrete decks. Due to the Polish law at the time of the design and construction of the bridge, a separate deck structure had to be installed under each roadway. The decks are 18.57 m wide and 2.50 m high, suspended to one pylon with 160 stay cables (Figure 2). The stay cables are divided into four equal planes. The theoretical cable lengths range from 67.39 m to 267.34 m. Each cable consists of seven-wire strands, which are installed from 24 to 48 strands in a bundle depending on the stay cable. 

**Figure 3:**
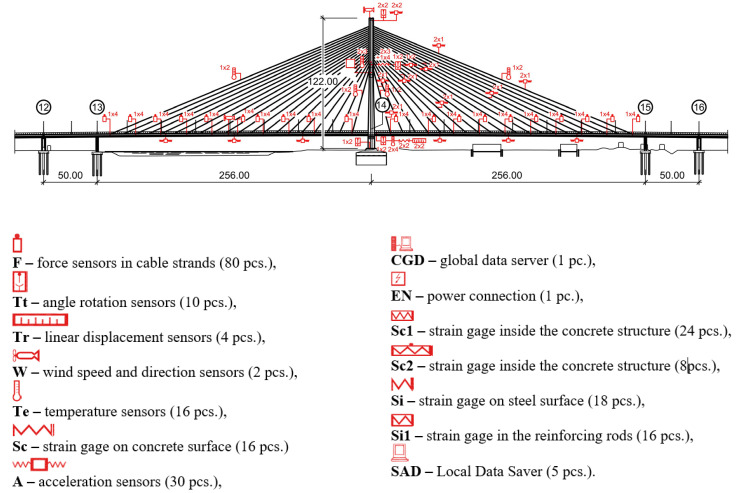
The structural health monitoring (SHM) system scheme [6].

Detailed stay cable parameters are presented in Table 1. The size and outstanding structure of the Rędziński Bridge were the reasons why it was equipped with 222 structural health monitoring (SHM) system sensors (Figure 3). A detailed description of the design and construction of the bridge can be found in the study [7].

The installed sensors are measuring the following values: stress, temperature, accelerations and displacements [6]. The connection with the system is allowed by an Internet browser application that provides an overview of each sensor. It generates alerts and notifications if some sensors are out of order. Furthermore, the application makes it possible to create diagrams of measured values and to export them as .csv files which are compatible with calculation programs. After 7 years, a database of the measured values was compiled, and it was compared with calculations conducted based on the bridge FEM model.

In case of the Rędziński Bridge SHM system, mostly vibrating wire strain gages were used. Measurement of all static quantities is performed at the same time, with the possibility of changing the time interval between readings from 10 to 60 min. Dynamic quantities (accelerations) and forces are measured with a frequency of 100 Hz. Their signals are sent to six local servers (SAD). Then they are saved on hard drives and sent to the data collection centre (CGD) located in the motorway management centre. In the concrete and steel elements of the bridge structure, strains are measured and converted into corresponding stress values. System measurements are additionally supported by the continuous geodetic measurement of pylon foot settlements.

Each sensor has a so-called address. It is a code that indicates the place of its installation in the structure, the type of measured quantity and the method of measurement. Table 2 shows the method of marking the sensors on the platform, pylon and cables. The sensors measuring the values in the cables are marked with the letter W. The cables are numbered from the pylon to the span direction. Additional markings with the letters L and P indicate the left and right deck of the structure. The letters W and Z indicate the stay cable attachment to the inner or outer edge of the deck, respectively. Table 3 presents a list of sensors used in the bridge.

Sensors measuring the tension force are only installed on the stay cables reference strand. The isotension method used for the installation and tensioning process attempts to create uniform stresses by applying certain sequence and force applications to individual strands. The uniformity of the strand forces was checked by an individual strand lift-off after each installation at the quality control step [8].

The relationship between the measured frequency by the vibrating wire strain gages and the deformation (strain) is described by the Formula (1). The fundamental frequency (resonant frequency) of the vibration of a wire is related to its tension, length, and mass:

(1) $$f=\frac{1}{2L}\sqrt{\frac{F}{m}}$$

After the transformations, the following equation may be obtained:

(2) $$\varepsilon =\frac{4mL^{2}f^{2}}{EA}=\frac{4\rho L^{2}f^{2}}{E}$$

where:

*f*—frequency (Hz), *L*—wire length (m), *F*—wire tension force (N), *m*—wire mass per unit length (kg/m), *ε*—strain (-), *E*—Young’s Modulus (Pa), *A*—wire cross section area (m^2^), *ρ*—wire material density (kg/m^3^).

Equation (1) can also be used for the determination of tension forces in stay cables. This approach was described in [9] and its usefulness for future research is discussed in Section 9.

## 3. The FEM Models

**Figure 4:**
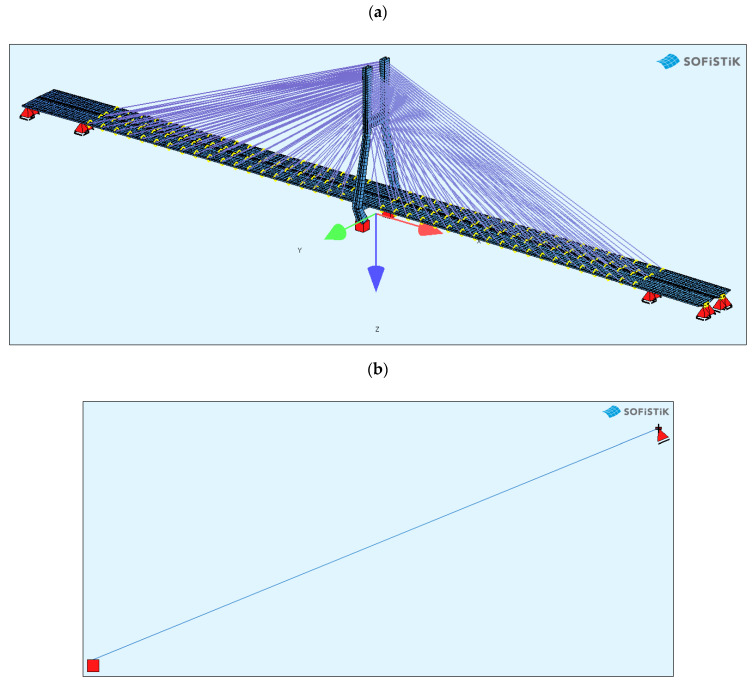
The FEM models: (**a**) the general bridge model; (**b**) a detailed single strand model.

The bridge FEM models were created in the SOFiSTiK software (Figure 4) by using the documentation provided by the bridge designer. For a general analysis, a whole bridge model was created. The pylon and deck elements were modelled as beam elements, but real cross-sections, not only the geometric characteristics, were used as the input for the model. The steel core in the upper parts of the pylon was modelled in the specified sections, because SOFiSTiK enables the calculation of the geometric characteristics just as in the case of a substitute cross-section.

The bridge stay cable system is made of Freyssinet cables [8]. Each cable is composed of a different number of strands (from 24 to 48). For the sake of simplification, the cables were modelled as circular cross-sections with an area corresponding to the number of those strands. In the general model, the cables are modelled as a single element, which can only be tensioned. In this model, all calculations were conducted according to the linear analysis.

In order to test the exact work of a single strand, a representative strand model was developed for each cable (Figure 4b). In this case, both a non-linear model of geometry and material was adopted. The cross-section was described by the parameters for a seven-wire strand. The loading on such a strand was its own weight and the tension force obtained from the general FEM model or from the SHM system. The model was discretized with elements from a length of 0.1 m. The verification of such a model was the overhang, calculated on the basis of its own weight and the average tension force, which in the case of the longest stay was 1.20 m. This value was consistent with the design assumptions. Such a model will be used in the future to assess the durability of strand anchorages where a bending moment appears, depending on the tension force.

**Figure 5:**
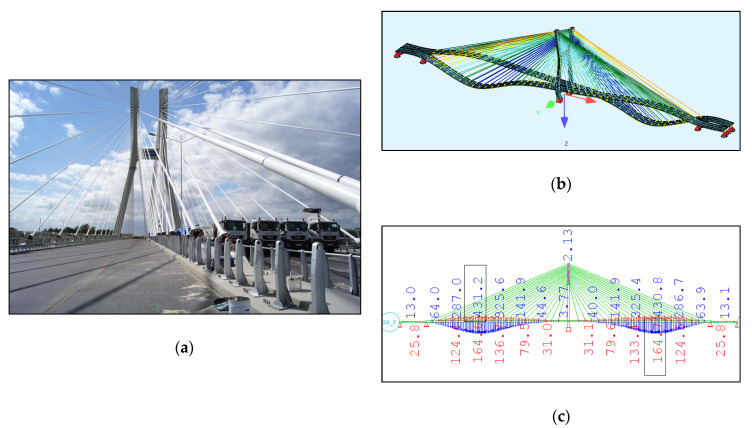
(**a**) The bridge during loading test [10]; (**b**) FEM model loaded; (**c**) corresponding loading deck nodes displacements in mm.

To be sure that the general model was created correctly, it was loaded with the load that was used during the static and dynamic load tests, described in [10], i.e., with the load of twenty 40-tonne lorries on each deck. The displacement of the FEM model is shown in Figure 5 and is very close to the load tests values. To compare, during the static proof-load test, a displacement of 467.0 mm was measured. The calculated displacement totalled 431.2 mm. During the test loads, several different load schemes were used and compared with the model. This article presents a comparison with a representative result. Furthermore, the first five frequencies of the natural vibrations were calculated and compared in Table 4. After those numerical tests, the FEM model was used for the calculations based on the SHM data.

## 4. Comparison of the Measured Data

### 4.1. Temperatures

**Figure 6:**
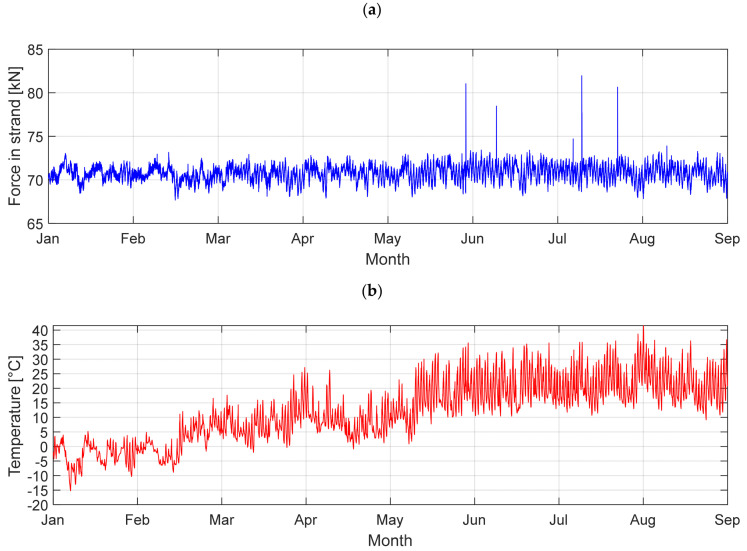
The diagrams in the longest cable LW-40: (**a**) force diagram; (**b**) temperature diagram.

The analysis of the temperature change’s influence on cable forces was started with the comparison between annual summer and winter values. Diagrams from the SHM application showing the force and temperature plots in one of the longest cables between January 2017 and August 2017 are presented in Figure 6.

Table 5 shows the temperature differences in the bridge parts (pylon, deck and cables) which were recorded by the SHM system. Those changes were applied at the FEM model as a constant increase in temperature for individual structure elements. The results of the calculations are shown in Table 6. The measured force differences in strands between the coldest and warmest days in 2017 are described as Δ*F* (SHM). The calculated force change is described as Δ*F* (FEM).

Comparing the calculated and measured values, it is visible how much they differ from each other. This indicates how complicated the temperature distribution problem is concerning bridges [11] or other civil engineering structures [12,13]. The SHM bridge system measures these values at a few selected points, which gives only a local view of these changes, and does not characterise the entire temperature distribution in the whole structure. Therefore, these measurements cannot be used for global or long-term structure analyses.

**Figure 7:**
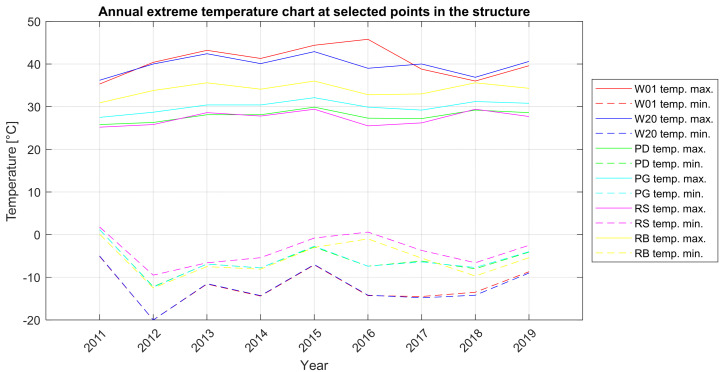
Annual extreme temperature chart at selected points in the structure.

In order to obtain a wider description of the nature of the work of the bridge structure, the changes in extreme annual temperatures in individual elements of the structure were additionally checked (Figure 7).

Characteristic points in the structure were adopted for the thermal analysis:

- the longest stay cable (symbol W-20),
- the shortest stay cable (symbol W-01),
- bottom of the deck section (symbol PD),
- top of the deck section (symbol PG),
- steel in the pylon’s upper cross-beam (RS symbol),
- concrete in the pylon’s upper cross-beam (symbol RB).

**Figure 8:**
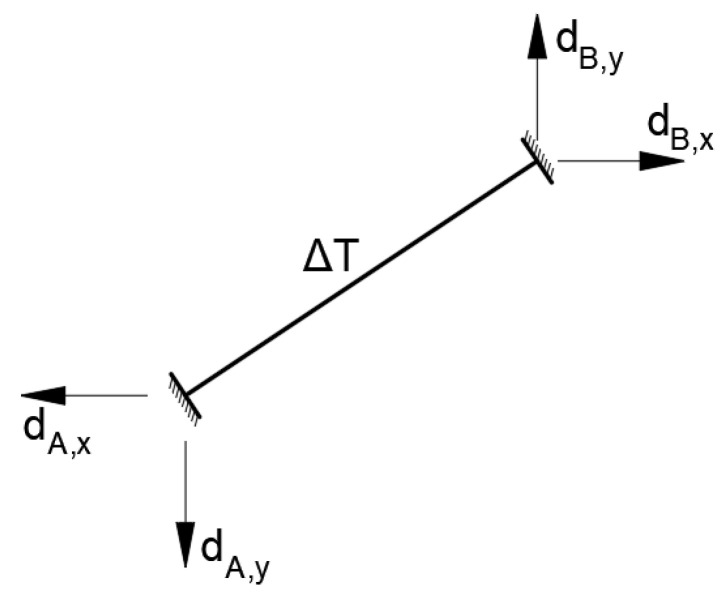
Strand loading scheme: Δ*T*—uniform temperature change; *d_i,j_*—theoretical anchorages displacements.

However, the measured temperature values can be used for the local calculation of structural elements. For this purpose, the measured temperature changes were used to calculate a single strand in the FEM model. This model, as mentioned, is described by the nonlinear material model and geometry characteristics as well. The crucial value in these calculations was the starting daily strand tension force. Then, a uniform temperature change Δ*T* was applied to the strand. Additionally, displacements of the theoretical anchorages, resulting from heating or cooling in the global bridge model, were implemented (Figure 8). The calculations were performed by automatic iterations, where the temporary structure stiffness was saved after each step. The calculations ended when the stiffness and cable sag values had been stabilized.

**Figure 9:**
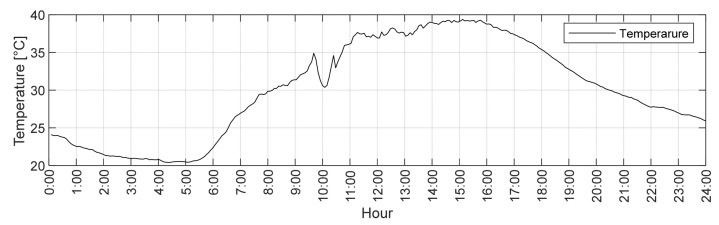
Daily strands temperature change (1 August 2017).

**Figure 10:**
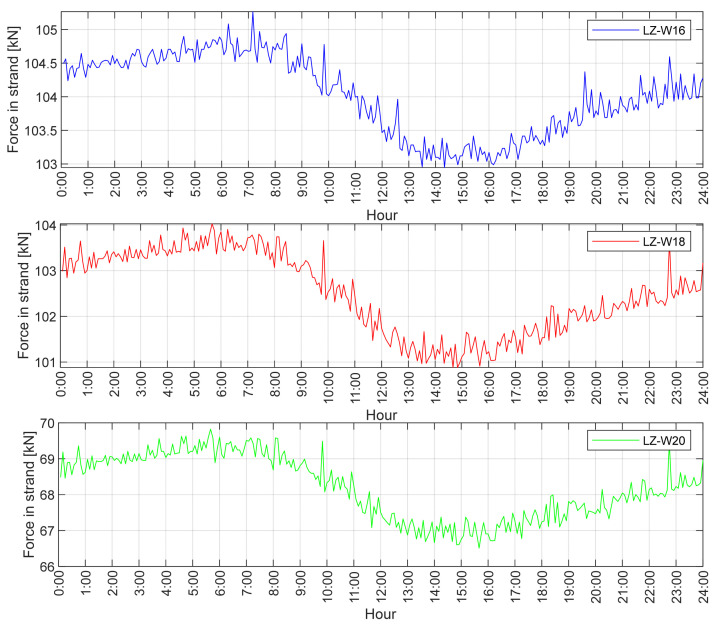
Daily force course in single strands of cables LZ-W16, LZ-W18 and LZ-W20 (1 August 2017).

Table 7 shows the described calculation results for the three longest stay cables. Based on the graphs in Figure 9 and Figure 10, the range of the maximum and minimum force as well as the temperature changes were determined. Due to disturbances in the measurement caused by vehicle traffic, it was decided to present the extreme values as a value range. It is worth noting that between 10:00 and 11:00 a.m., there was a rapid temperature change by about 4 °C, which resulted with a force peak at the same time. As for the value, a similar force change occurred between 0:00 and 6:00 a.m. by the temperature difference of 3.7 °C.

The presented analysis and results comparison show that a detailed calculation approach accurately reflects the work of the structure in the context of the temperature changes of cable elements. The temperature changes are significant in relation to the displacement of the cable anchorages relative to each other. Applying them in the cables detailed in the FEM model allows for a more accurate estimation of force changes and stresses in particular cable sections. However, the analysis of the temperature distribution in whole suspended structures composed of various materials is a complex issue and requires further and more accurate measurements than just SHM-based measurements to be able to create a representative load model. A complex description of temperature distribution in bridges is presented, e.g., in [11]. It is also worth adding that the differences in temperature distribution, that should be applied to the calculation models, are included in the design standards. The analysis performed according to SHM measurements, supported by FEM calculations, allows to check the plausibility of the standard assumptions.

### 4.2. Traffic Loads

**Figure 11:**
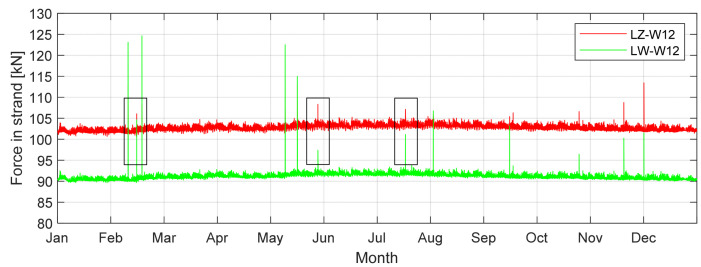
Force diagram in the middle-length cables LZ-W12 and LW-W12 with selected maximum force peaks in 2017.

**Figure 12:**
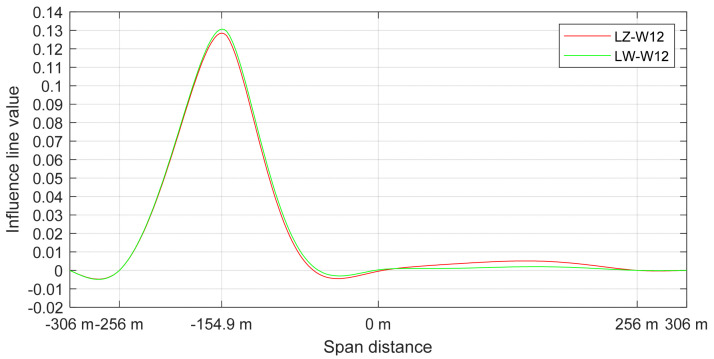
Influence line of tension force for cables LZ-W12 and LW-W12.

Figure 11 is a diagram for the cable force in the year 2017 in one of the medium-length cables. Major force peaks occurred on the diagram in February, May, July, October and December. This was the reason for creating influence lines for the cable tension force (Figure 12). Table 8 shows calculations that were helpful in the assessment of what kind of vehicles may have crossed the bridge. A force sensor installed on the cable only measures the force in one single strand. For the force assessment, it is necessary to know how many strands are installed in each cable. Multiplying the force results from the SHM measurement by the number of strands is allowed due to the cable assembling technology used. It provides the same stress in each strand [8].

Some of the peaks on the force diagram may be considered to be measuring errors. In order to exclude them, it is necessary to check whether the neighbouring cables have similar peaks at the same time. Table 8 presents estimations for the proved peaks only (also marked on Figure 11). The bigger peaks cannot be caused by a single vehicle, so it was necessary to consider a situation with a traffic jam or the accumulation of heavy vehicles. The situation is presented in the last column of Table 8.

**Figure 13:**
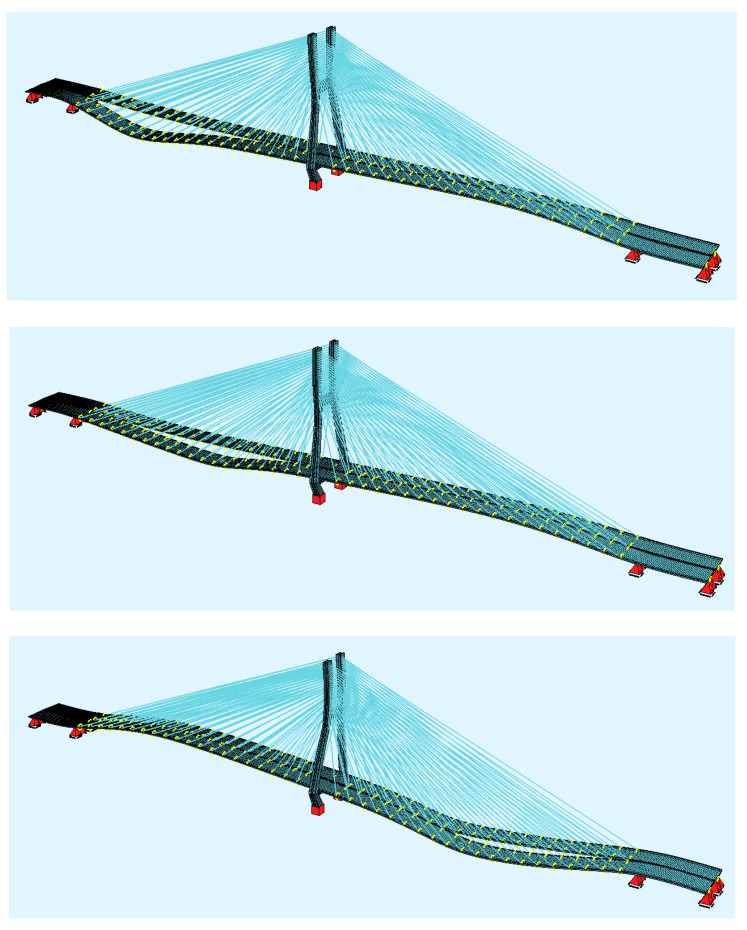
Screenshots from the moving vehicle on the FEM bridge model.

**Figure 14:**
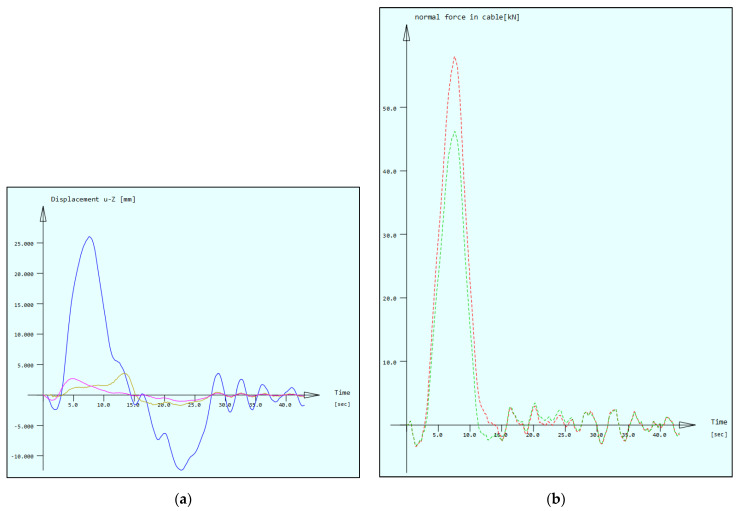
(**a**) Node displacements for 3 measuring points on one span, magenta line: deck point under the longest cables W40, dark blue line: deck point under the medium–long cables W32, brown line: deck point under the shortest cables W21; (**b**) tension force in the whole cables W12, red line: outer deck cable, green line: inner deck cable.

After the assessment of crossing cars, a simulation of a moving vehicle on one bridge deck was carried out with the FEM model. The aim of that calculation was to assess the biggest deck displacement and accelerations during the crossing and to check the medium force peaks appearing on the cable force spectrum. The calculation was carried out for a 40-tonne truck that was moving with the speed of 80 km/h (Figure 13). Figure 14a shows the vertical deck displacements for three points of the deck structure (description under the figure).

The SHM system is not equipped with vertical displacement sensors. With a proper FEM model, the bridge supervisor is able to assess that important value. The biggest displacement was noticed in the middle part of the span (26.05 mm). Figure 14b shows the force change that appeared in the medium-length cables W12. There can be noticed a difference between the inner and outer cables. The outer cables are a little longer, because of the deck rotation to provide the transverse slope on the road surface. It causes an unsymmetrical load distribution between cables. Such a difference is also noticed by the SHM system (Figure 11) in the corresponding cable pair.

Table 9 shows the calculation of force change for the LZ-W12 and LW-W12 cables. The values were calculated for a single strand (these particular stays consist of 48 strands each). It was assumed that one vehicle weighing 40 tonnes is moving on the deck. The results show that for a simple and quick estimation of the tension force changes, it is enough to use the tension force influence line. For a more detailed description, a complete analysis of the vehicle’s crossing should be confuted, because then, the work of the stay cable system is more accurately included. As a result, the difference between the force change in the external and internal cable is noticeable. This relationship is also visible in Figure 11, where the measured force also takes into account the self-weight of the structure. For larger vehicles passing, different peaks are visible.

The presented and verified approaches for estimating changes in tension forces can be used to characterize the work of cables that are not equipped with sensors.

**Figure 15:**
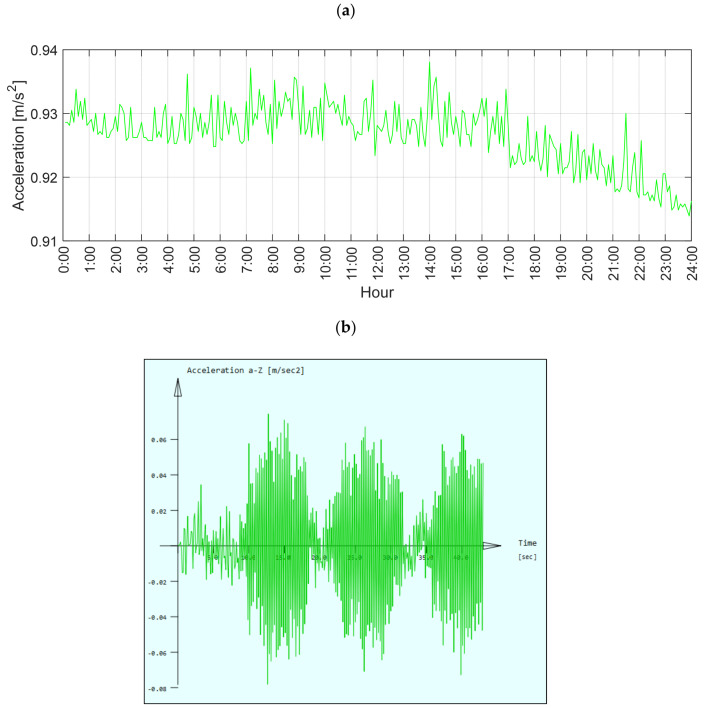
(**a**) Deck accelerations near the cable W-32 anchorages registered by the SHM system; (**b**) vertical acceleration in the middle span deck point (below the cables W-32) during the vehicle crossing.

Another important issue is the estimation of the vibration acceleration of the structure on the basis of vehicle passage. Although the SHM system is equipped with vibration sensors, these do not give accurate results as they record the measured values every 5 min. For dynamic values, such a time interval is completely inefficient. It only gives information about the range of changes in the vibration acceleration of the structure. Both phenomena are presented in the daily measurement of the acceleration of the vertical vibrations of the deck at the anchorage of the W-32 cables. Note that the system acceleration sensor requires calibration; changes oscillate around a value other than zero (Figure 15a).

Figure 15b shows the results of the vertical accelerations in the middle span deck point. The maximum accelerations are between 0.06 m/s^2^ and 0.08 m/s^2^ and are the effect of a heavy vehicle crossing. Lower calculated values (0.02 m/s^2^–0.04 m/s^2^), before the crossing, correspond to the values change range measured with the SHM system. Accelerations of the vibrations at this level are negligible. The calculation model assumes no damping. The damping factor for such a structure as the Rędziński Bridge should be tested separately, which is a subject of ongoing investigation. The presented approach aims to show only the maximum possible values of vibration accelerations, which supplement the data measured by the SHM system. Further advanced static and dynamic analyses should be carried out in accordance with [14,15].

The comparison shows that due to the required sampling frequency in measurements of the vibration acceleration, long-term monitoring (several years) would be too burdensome. For this purpose, high-capacity, high-speed storage drives would be needed, which from a maintenance point of view, could prove to be an expensive undertaking.

The peaks appearing on the force diagrams in SHM data often approach the upper safety limit set in the SHM system, therefore the cause of their appearance should be determined each time. Often, these can be just a measurement error. However, when they are a real representation of the load and occur too often, they can cause fatigue damage to cables. Therefore, their source should be determined, and the effects of this load checked in FEM models. It should also be remembered that exceptional vehicles often pass on the bridges and need the appropriate permits. The manager equipped with the SHM system of the facility and its FEM model can, using such tools, easily determine the conditions and possibilities of such a passage and what impact it may have on the durability of the structure. In addition, we are constantly dealing with an increase in vehicle traffic on the roads. The ongoing updating of calculations compared with the results from the SHM system will allow for the accurate determination of the current load capacity of the object. When the calculated and measured values start to differ significantly, this will indicate a possible failure of the facility and a quick decision may prevent a catastrophe.

## 5. Cable Forces Analysis

From the design and analytical point of view, it is important to determine the impact of individual types of loads on the fatigue strength of stay cables. The works [6,16] discuss various approaches to considering the moving loads on bridges. It is assumed that continuous vehicle traffic has a completely different impact on the forces and displacements (vibrations) of cables than other short-term intense weather changes such as windstorms or big amplitudes of temperature. The amount of collected data allows one to create statistics on the occurrence of particular types of bridge loads. An additional advantage of the monitoring system measurements is the fact that the data are collected continuously in real time. Parallel simulations based on the already collected data can be verified on an ongoing basis. The measured tension force in the strands is the effect of various impacts on the bridge, which include dead load, thermal impact, vehicle crossing and wind pressure on the structures. The whole impact can be described with the general formula:

*F*(*t*) = *g*(*t*) + *T*(*t*) + *q*(*t*) + *w*(*t*), (3)

where:

*F*(*t*)—total force in the cable strand; *g*(*t*)—component of force from dead load; *T*(*t*)—component from thermal interactions; *q*(*t*)—random function that illustrates the impact of passing vehicles; *w*(*t*)—random function representing the influence of wind; *t*—time.

Comparing the diagrams in Figure 9 and Figure 10, the influence of temperature on the change of the tension force is clearly visible. The temperature drop leads to increased tension in the stay cable and vice versa. Analysing the fluctuations of forces during the day, one can ignore the influence of rheological changes in the concrete of the bridge and the pylon. This change, in the form of function *g*(*t*) in Equation (3), is negligibly small, and only noticeable in the long-term analysis.

For this reason, in the first phase of the analysis presented, Equation (3) will be simplified to:

*F*(*t*) = *G*(*t*) + *Q*(*t*), (4)

where:

*G*(*t*)—total tension force from own weight and temperature changes; *Q*(*t*)—changes in the tension force from variable loads. Function *G*(*t*) was approximated by successive series of the Fourier series:

(5) $$G\left(t\right)=a_{0}+\overset{n}{\underset{i=1}{\sum }}a_{i}cos\left(i\omega t\right)+b_{i}sin(i\omega t)$$

The correctness of fitting the *G*(*t*) function to *F*(*t*) was measured with four parameters.

Sum of Squares Due to Error. This statistic measures the total deviation of the response values from the fit to the response values. It is also known as the summed square of residuals and is usually labelled as SSE. The closer the value is to 0, the better the match.

R-Square. This statistic measures how successful the fit is in explaining the variation of the data. In other words, R-square is the square of the correlation between the response values and the predicted response values. It is also known as the square of the multiple correlation coefficient and the coefficient of multiple determination. The closer the value is to 1, the better the match.

**Figure 16:**
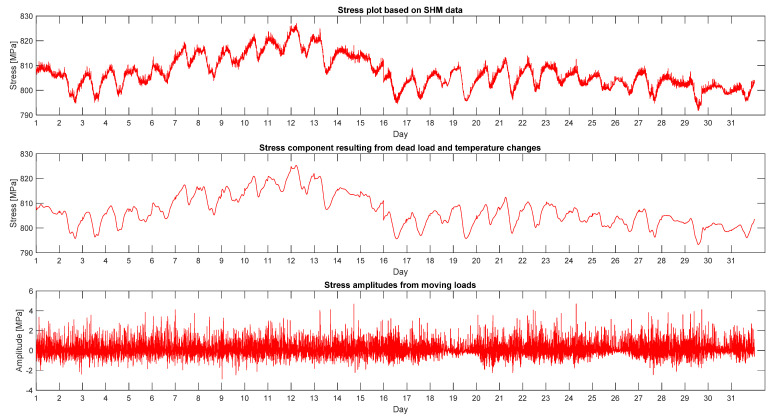
Graphs showing split data according to the described algorithm.

Root Mean Squared Error. This statistic is also known as the fit standard error and the standard error of the regression. It is an estimate of the standard deviation of the random component in the data. The closer the value is to 0, the better the match. Table 10 presents an example of the scope of the particular fit parameters for subsequent stays on 17 August 2018. Figure 16 presents the result of such an operation for one whole month (May 2013) for the W16 stay. The first graph is a pure data plot from the SHM system, *F*(*t*), the next is a graph of successive approximated daily functions, *G*(*t*), and the last one shows filtered force changes—amplitudes, *Q*(*t*).

On the basis of such mathematical operations, monthly and annual histograms describing the range of stresses and their changes were made for the separated graphs. Their statistical interpretation will be presented in the next chapter. The presented algorithm can also be an alternative or supplement to the commonly used rain-flow cycle counting method. The basics of such probabilistic calculations are described, e.g., in [17,18].

The presented mathematical procedure allows for a better understanding of the characteristics of the structure’s work. According to the authors, subsequent SHM systems could be equipped with such, or even more complex, algorithms for an immediate separation of the current measured signal.

## 6. Histogram Interpretation

**Figure 17:**
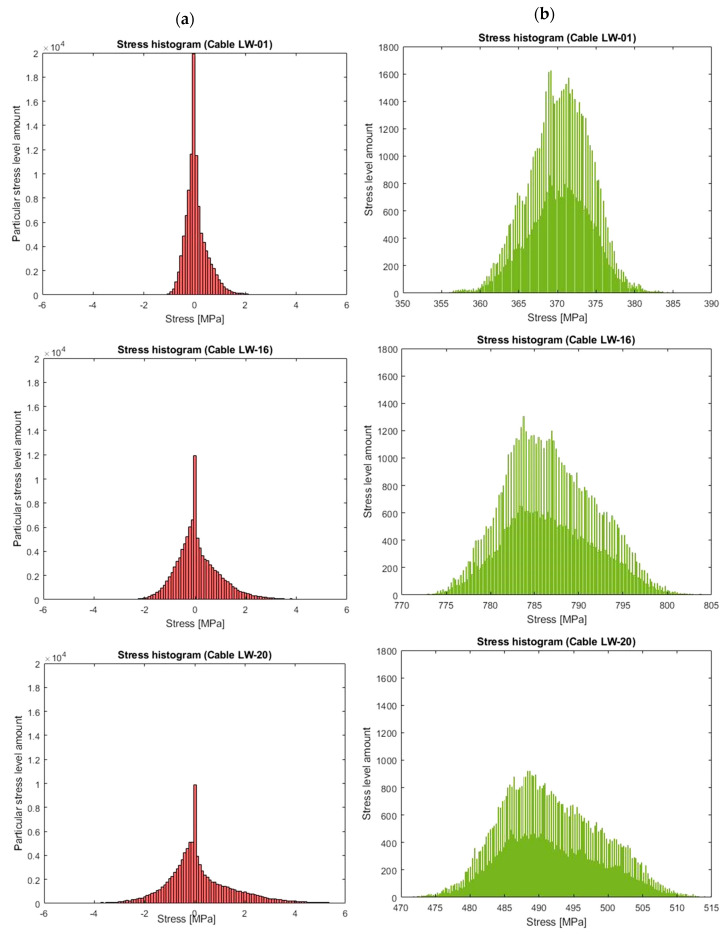
(**a**) Histograms of stress changes.; (**b**) amplitude histograms without separated temperature influences.

Figure 17a shows these histograms for the discussed cables. Ranges from −5 to 5 MPa were defined in order to improve their readability. By examining the entire stress spectra, stress changes of up to 60 MPa appear in them. Figure 17b presents the histograms of stresses in the analysed cables without separating thermal influences and dead weight. The dissipation of stress values over a year is visibly larger due to the influence of temperature on the tension force in the cable.

According to the Palmgren–Miner rule, each cycle *n_i_* (two amplitudes are a whole cycle) causes a small damage in the steel structure *n/N. N* is the amount of particular destructive cycles due to the Wöhler curve and *n* is the cycles measured. More extensive descriptions of the advanced fatigue calculations, considered in this article, can be found in [19,20]. If *D* describes the sum of all damages caused by different cycles, a simple equation can be formulated, namely:

(6) $$D=\overset{q}{\underset{i=1}{\sum }}\frac{n_{i}}{N_{i}},$$

In order to compare this classical method of counting cycles, comparative calculations were made to the approach proposed in this paper. Two comparison parameters were introduced: *D_a_* and *D_s_*.

(7) $$D_{a}=\overset{n}{\underset{i}{\sum }}\sigma_{a,i}n_{i}$$

(8) $$D_{s}=\frac{1}{2}\overset{n}{\underset{i}{\sum }}|\sigma_{s,i}|n_{i}$$

At this point, Formulas (7) and (8) should be interpreted. Based on the Formula (6), parameter *n_i_* was omitted. This procedure is allowed due to the fact that *n_i_* is a constant value resulting from the *i-th* interval directly from the Wöhler curve. The magnitude of $\sigma_{a,i}$ is the amplitude of one complete symmetric cycle of change in stress, calculated with the rain-flow procedure. These are values greater than zero. Coefficient *D_s_* contains stress $|\sigma_{s,i}|$ oscillations around the average value. Due to the method adopted here, this value is 0, and therefore the fluctuations are negative and positive. They can be treated as separate upper and lower half cycles. Therefore, value 0.5 appears before the sum sign in Formula (8) to enable a comparison with parameter *D_a_.* Table 11 compares parameters *D_a_* and *D_s_* for the analysed stay cables from 2018.

As can be seen, ratio *D_s_/D_a_* is greater than zero. On this basis, it can be concluded that the changes in the forces in the stays are not symmetrical. Tension is greater than compression.

The histograms created are an introduction to performing accurate fatigue calculations. The methods described in the literature [20,21] are based on the constant mean force, the accompanying amplitudes and the Wöhler curve. The above analyses show that with long-term observations, the mean tension force changes. Therefore, it is important to separate the long-term and short-term effects, and the calculation of the failure accumulation parameter *D* should be performed for smaller data ranges, e.g., by weeks or months. Even if the parameter *D* will not increase quickly, it can be used in further calculations as an index reducing the load capacity of the material due to microscopic damage to the material structure. Moreover, the above analysis of the stay cable statics shows that the classical fatigue strength calculation methods for cables, based on the Wöhler curve, may be inaccurate. Therefore, the so-called fatigue surfaces or Haigh diagrams should be used as it is indicated in [18,21,22]. Their methodology is based on a separate influence of the mean force and amplitudes, which solves the problem of the differences between the presented parameters *D_a_* and *D_s_.*

**Figure 18:**
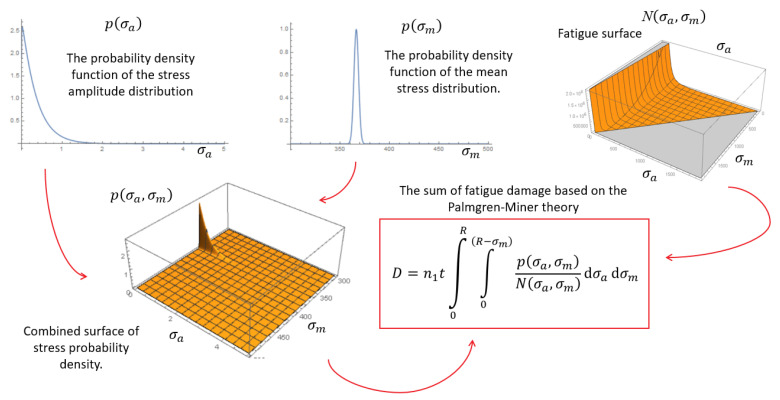
Calculation scheme of the fatigue parameter *D*, based on the fatigue surfaces [18,21,22]. Description of markings in the text.

The general idea of calculating the *D* parameter on the basis of the fatigue surface is shown in Figure 18. In order to create such a surface, two data distributions should be used: *p*(*σ_a_*)—the probability distribution function of amplitude stress changes, and *p*(*σ_m_*)—the probability distribution function of the average tension stress. The functions describing these distributions are presented in Table 12. They were calculated on the basis of the separated diagrams shown in Figure 16 and Figure 17.

The fatigue surface *N*(*σ_a_*, *σ_m_*) is calculated and described individually for each steel, just like the Wöhler curves [18]. The stress probability density area *p*(*σ_a_*, *σ_m_*) is the product of the single probability distribution functions (PDFs), described above. *R* is the yield strength of steel; *n*_1_ is the number of cycles in the measured period *t*. The whole idea is derived directly from Formula (6).

Table 13 shows the calculated parameters for measurements from the available years and for future data simulations, in accordance with the projected durability of the particular bridge. The method of data simulation is described in Section 7.

## 7. Data Simulations

**Figure 19:**
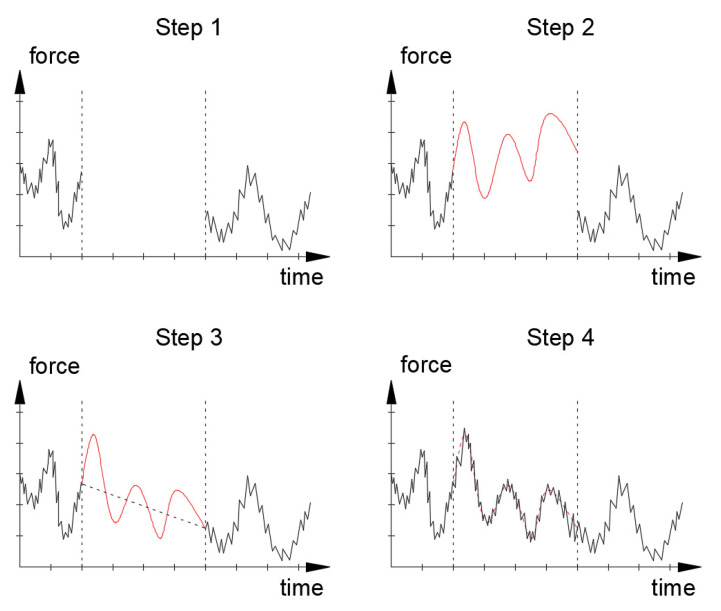
Data simulation scheme. Step 1: no data; step 2: simulating the average course of forces on the following days on the basis of a randomly selected Fourier series from the created database; step 3: linear data fit correction; step 4: simulation of amplitudes using the inverse distribution function.

A frequent problem of large monitoring systems are their temporary failures related to electricity supply, etc. This may result in longer or shorter periods related to the lack of measured data. To do this, in order to keep the overview, the data should be properly simulated. The advantage of long-term SHM measurements is that their database is constantly growing. The histograms and graphs created above show how data can be divided and sorted. In such a prepared form, they can be used to simulate consecutive or missing data. Figure 19 shows the idea of such a simulation scheme.

**Figure 20:**
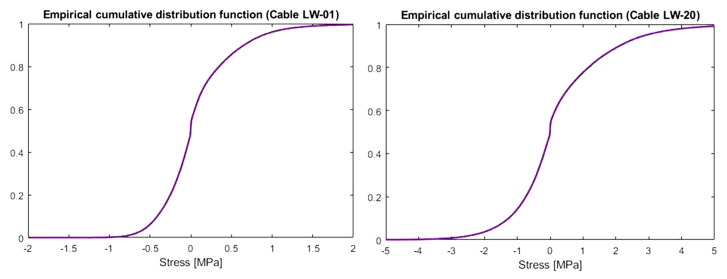
Empirical cumulative distributions for stress changes in the shortest (LW-01) and longest cable (LW-20).

Using data from the relevant months in the previous years, an empirical distribution of the stress change distribution can be created (Figure 20). Additionally, a matrix containing all approximated functions (Fourier series) is described by Formula (5). The simulation began with a random selection of successive polynomials describing the mean force value. This fit was next adjusted linearly to obtain signal continuity. The last step was to add random amplitudes to which the inverse cumulative distribution method was applied, using the empirical cumulative distribution function just created. All calculations were made in the Matlab software according to original scripts.

**Figure 21:**
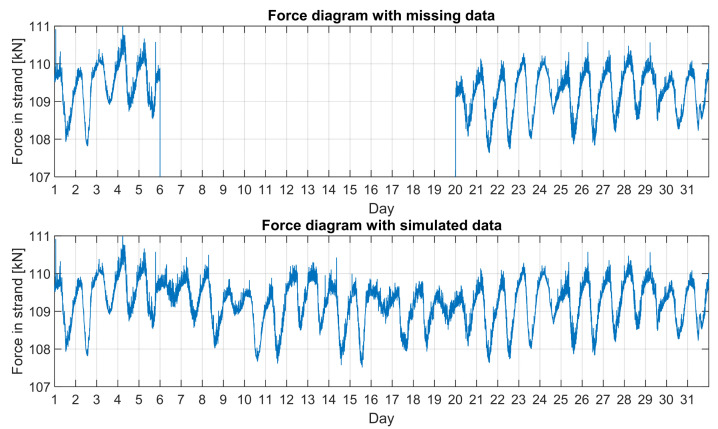
LW-16 force chart in July 2016. Upper chart with missing data and lower with simulated one.

Figure 21 shows an example of a graph with missing and simulated data parts obtained by using the method specified in Figure 19 and Figure 20. The chart obtained in this way can be used for further theoretical analyses. In a similar way, assuming different levels of the average tension of the cables, a series of data simulations for subsequent years were performed. In this way, *D* parameters were calculated according to the formula in Figure 18. Table 13 summarises the calculated annual *D* parameters for measured and simulated (years marked with *) data, respectively. First, monthly values were calculated and summed up over the following years.

From the results in Table 13, it is clear that the bridge was designed correctly, and the stay cables are not exposed to fatigue damage from changes in tensioning forces. Another sensitive point may be their anchors, where the influence of cable bending should also be considered [3,4,5]. This is a separate research topic that is to be the subject of future analyses. However, the data show that the greatest *D* parameter increment occurs in the LW-20 cable, with the largest force/stress amplitude, and in the LW-16 stay with the highest average tension force.

## 8. Characteristics of the Influence of Individual Loads on the Cable Tension Force

The analysis presented above shows how differentiated the influence of individual loads is on the total tension force in stay cables. For the concrete cable-stayed structure, which is the Rędziński Bridge, its main component comes from its dead load.

Based on the observations, a cyclic change in the tension force in the cables was found. It increases in the summer and decreases in the winter. Similar fluctuations occur in the cycle of daily changes, with the difference being that during the day, there is a decrease in strength and an increase at night. This phenomenon is a result of the deformation of the pylon and the deck due to temperature changes. In daily cycles, the tension force is affected by the deformation of the superstructure itself, while the difference in the mean tension force between the summer and winter results from the mutual displacement of the anchorages on the pylon and decks. Local and rapid changes in the tension force are caused by the passage of heavy vehicles or by the overlapping effects of individual moving loads. These types of effects are noticeable within the entire group of stay cables, located in the load zone of the decks.

The authors realize that these are not all of the factors influencing the long-term stress of cables in cable-stayed bridges. The influence of rheology on force redistribution, pylon settlement [23] or the complicated issue of aerodynamics or seismic issues [24,25] remains a problem. However, in this paper, the aim was to initially decode the values measured by a specific SHM system, which in the coming years will provide a lot of information that could complement the analysis that has already been started.

## 9. Discussion

Monitoring and continuous observation of bridge structures has become an important part of the work of designers and scientists over the last few decades [1,7,15,17,26,27,28,29,30]. Both new structures and old ones require supervision for maintenance purposes [31]. Structural health monitoring (SHM) allows one to monitor the structure according to its purpose: old bridges should be monitored in the places of identified damage [32], and the examination of new bridges enables a real assessment of the structure’s behaviour. SHM system saves a lot of information from a big number of sensors. The analysis of the results should be approached with caution. First of all, on the basis of the measurements, the behaviour of the whole structure should be assessed, and then it should be ensured that the results are reliable. For this purpose, it is worth creating an auxiliary FEM model of the tested object to verify the measured data, which has been shown in this paper. Measurements only carried out with SHM systems, or theoretical calculations alone, do not allow a good verification of the bridge behaviour and the assessment of its condition. Furthermore, the load test results of the Rędziński Bridge provided additional information to verify the FEM model. Such a set of available research tools and methods can be useful for assessing the durability of the bridge structure and its elements. However, continuous monitoring with the support of SHM systems and data verification is a reliable source of information about the behaviour of cable-stayed bridges, but a detailed analysis of data from SHM also allows one to create an extensive statistical database. By adding calculations based on realisable FEM models, unmeasured SHM values can be defined. The calculation of the *D* parameter based on the available statistics and the implementation of advanced simulations ultimately allows one to develop a trend of the force changes influence on the stay cable system.

It can be stated that the creation of an efficient tool in the form of a coupled monitoring system with a numerical model can be easily used in practice. The analysis of the collected data presented in the article shows how complex the problem of determining the impact of individual loads on the effort of the structure is. In the case of structures with a complex, hyper static scheme, such as the described bridge, intuitive determination of the influences may turn out to be wrong.

**Figure 22:**
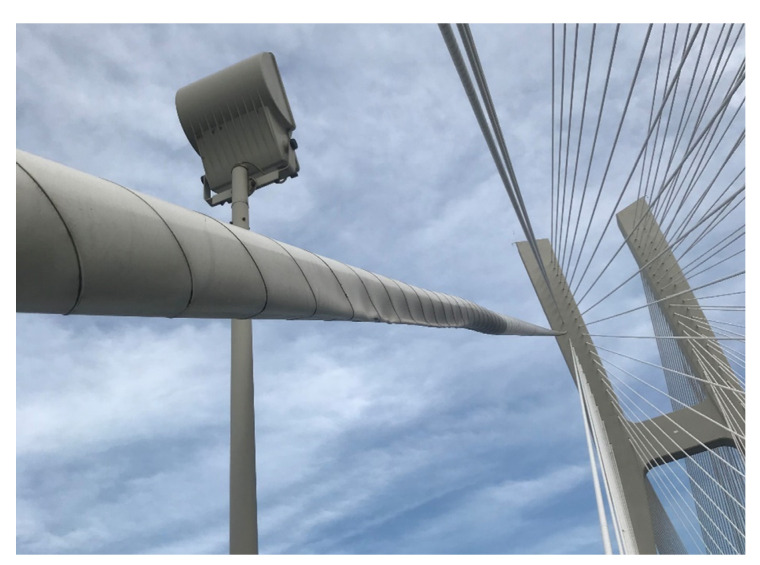
LZ-W11 cable sheath deformed as a result of a vehicle fire.

There are, however, exceptional situations that cannot be detected on the basis of SHM measurements. Figure 22 shows the deformed sheath of the LZ-W11 stay cable. This situation occurred as a result of a passenger vehicle fire near its bottom anchorage zone. Unfortunately, no force sensor is installed in this stay cable. However, checking the force values from neighbouring cables and their implementation into the numerical model allowed for the estimation of the force after the accident in the damaged stay cable. Ultimately, it could be concluded that, apart from the deformation of the cover, the internal strands were not damaged.

**Figure 23:**
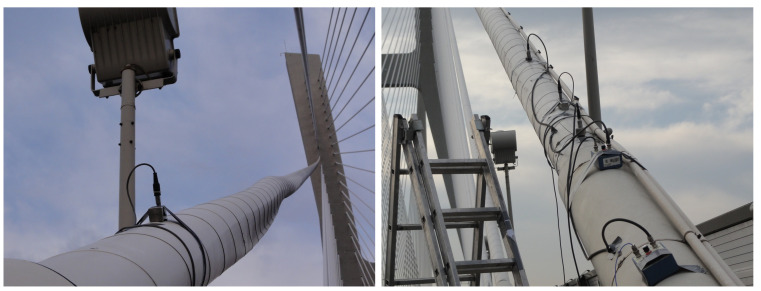
Accelerometers used for determination of a stay cable force.

An alternative method that can be used for the determination of a stay cable force are dynamic measurements by using, e.g., external accelerometers. The potential of this method is a subject of the Polish–Portuguese NAWA-FCT research project “Dynamic monitoring of bridge structures”, supported by the Polish National Agency for Academic Exchange and Fundação para a Ciência e a Tecnologia in Portugal, that is carried out, among others, on the Rędziński Bridge (Figure 23). Dynamic measurements indicate the wide applicability, easiness and practicality to validate the design assumptions of bridges and assess the condition of their stay cable systems [9,14]. This method can support the SHM system and the FEM model-based force analysis. It will be used as the future research direction on the Rędziński Bridge.

It is worth adding that the analysis of SHM data from the first 10 years of the bridge’s existence shows that it was designed correctly. Continuous observation of data from the monitoring system ensures the safety of the structure and its users, despite minor accidents such as a vehicle fire. Furthermore, the road administration (General Directorate for National Roads and Motorways) receives annual reports based on measurements from the SHM system supported by a constant FEM analysis.

## Figures and Tables

**Table 1:** Dimensions of stay cables and the number of strands installed in them.

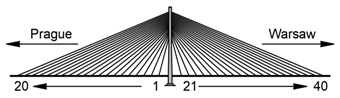			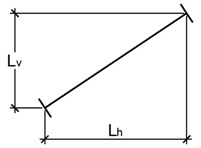		
Stay Cable Number		Number of Strands	Horizontal Length L_h_ [m]	Vertical Length L_v_ [m]	Total Length L [m]
1	21	24	19.06	64.64	67.39
2	22	28	30.51	67.58	74.15
3	23	28	42.33	69.94	81.75
4	24	30	54.29	72.03	90.20
5	25	32	66.30	74.07	99.41
6	26	34	78.31	76.01	109.14
7	27	38	90.37	77.95	119.35
8	28	40	102.41	79.85	129.86
9	29	46	114.45	81.75	140.65
10	30	48	126.51	83.62	151.64
11	31	48	138.54	85.52	162.81
12	32	48	150.57	87.41	174.10
13	33	48	162.61	89.29	185.51
14	34	48	174.59	91.16	196.96
15	35	48	186.63	93.06	208.55
16	36	48	198.66	94.97	220.20
17	37	48	210.70	96.90	231.91
18	38	48	222.75	98.84	243.70
19	39	48	234.79	100.77	255.50
20	40	40	246.82	102.71	267.34

**Table 2:** Examples of sensor markings.

Measured Quantity	Sample Mark	Description
Force in the stay cable	W16-LW/F	The sensor located in the stay cable No. 16 (W16), which supports the left deck (L) from the inside (W), measuring the tension force of the stay cable (F).
Temperature	W21-LZ/Te	The sensor located on the stay cable No. 21 (W21), on the left deck (L) from the outside (Z), measuring the temperature (Te).

**Table 3:** List and description of sensors.

No.	Description	Function	Photo *
1.	Vibrating wire strain gages integrated with temperature sensors: Geokon model 4000	Strain and temperature measurements in the pylon and decks	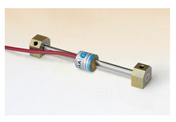
2.	Vibrating wire strain gages integrated with temperature sensors: Geokon model 4100/4150	Strain and temperature measurements in the pylon and decks	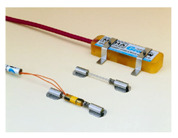
3.	Vibrating wire strain gages integrated with temperature sensors: Geokon model 4420	Measurement of linear displacements	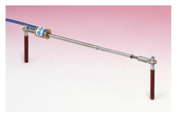
4.	Accelerometers: Haneywell model MA321	Measurement of structure accelerations	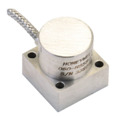
5.	Temperature sensors: Geokon model 3800	Temperature measurement of individual elements of the structure	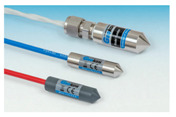
6.	Vibrating wire tiltmeters: Geokon model 6350	Measurement of angular displacements	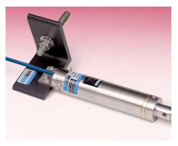
7.	Force sensor: Advitam Permanent Monostrand Load Cell Model MLC C P	Measurement of forces in stay cables	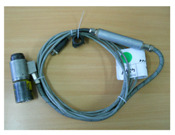

* Source of photos No. 1–6: https://www.geokon.com.

**Table 4:** Comparison of the calculated and measured frequencies.

Form Number	Frequency Number and Form	FEM [Hz]	Tests [Hz]
1.	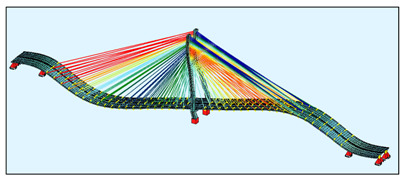	0.25	0.25
2.	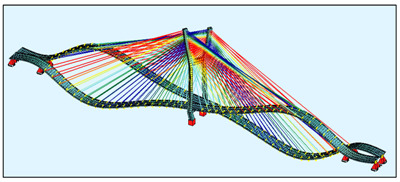	0.32	0.31
3.	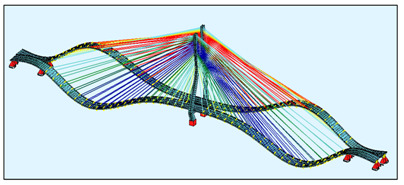	0.50	0.48
4.	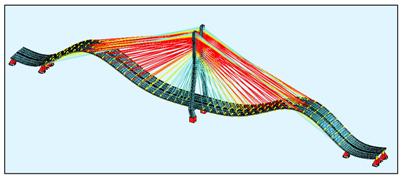	0.51	0.48
5.	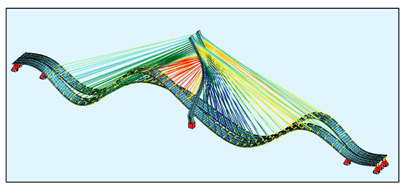	0.61	0.62

**Table 5:** Measured temperature differences in structure elements.

Element	Lowest Daily Average Temperature in January 2017 (°C)	Highest Daily Average Temperature in August 2017 (°C)	Δ*T* (°C)
decks	−2.40	22.90	+25.30
pylon	−2.40	22.80	+25.20
cables	−8.35	26.00	+34.35

**Table 6:** Measured forces in strands of selected (the shortest and longest) cables compared with the calculated tension force change.

Position	The Shortest Cable (LZ-W21) (kN)	The Shortest Cable (LW-W1) (kN)	The Longest Cable (LZ-W20) (kN)	The Longest Cable (LW-W20) (kN)
Highest daily average force in January 2017 (06/01/17)	63.14	51.82	73.17	68.31
Lowest daily average force in August 2017 (18/08/17)	62.80	51.04	73.04	67.91
Δ*F* (SHM)	−0.34	−0.78	−0.13	−0.40
Δ*F* (FEM)	−2.60	−2.63	−4.25	−4.21

**Table 7:** Comparison of forces changes between measured and calculated values on the basis of the single strand FEM model.

Cable Number	Initial Force (kN)	Maximum Force Range (kN)	Minimum Force Range (kN)	−Δ*T* (°C)	+Δ*T* (°C)	Calculated Maximum Force (kN)	Calculated Minimum Force (kN)
LZ-W16	104.5	104.7–105.3	102.9–103.4	−3.7	+15.3	104.91	102.78
LZ-W18	103.5	103.4–104.4	100.9–101.3	−3.7	+15.3	103.41	101.28
LZ-W20	68.5	69.3–69.9	66.5–67.2	−3.7	+15.3	69.31	67.18

**Table 8:** Selected and estimated force peaks in the year 2017.

Peak Number (Month)	Peak Value	Monthly Average Strand Force	Force Difference in Strand	Force Difference in Cable (48 Strands)	Estimated Loading on Whole Deck	Example Vehicles
1 (February)	101.8 kN	97.3 kN	4.5 kN	216.0 kN	360 t	3 loaded concrete mixers (3 × 40 t) + 10 lorries (10 × 15 t) and cars
2 (May)	103.6 kN	97.9 kN	5.7 kN	273.6 kN	460 t	Military transport (e.g., Stanag MLC 150) + lorries (15 t) and cars
3 (July)	105.0 kN	98.0 kN	7.0 kN	336.0 kN	570 t	Military transport (e.g., Stanag MLC 150) + lorries (15 t) and cars

**Table 9:** Comparison of tension force changes in one strand of LZ-W12 and LW-W12 cables calculated according to the influence line and with the modelled dynamic passage of a 40 t vehicle.

Stay Cable Number	Force Change Calculated from Influence Lines	Force Change Read from the FEM Model during the Dynamic Crossing
LZ-W12	1.07 kN	1.20 kN
LW-W12	1.05 kN	0.94 kN

**Table 10:** Sample parameters from 17 August 2018.

Cable	SSE	R-Sqr	RMSE
LZ-W20	1.2	0.984	0.067
LZ-W16	2.1	0.981	0.089
LZ-W01	5.4	0.976	0.141

**Table 11:** Parameters *D_a_* and *D_s_* in year 2018.

Stay Cable	*D_s_*	*D_a_*	*D_s_*/*D_a_*
LZ-W01	16,705.2	13,983.9	1.19
LZ-W16	34,320.3	28,593.4	1.20
LZ-W20	52,831.9	44,848.1	1.18

**Table 12:** Applied, exemplary types of probability distribution function of the analysed quantities.

	Probability Distribution Function (PDF)	Distribution Parameters
Average stress $\sigma_{m}$	Normal $p\left(\sigma_{m}\right)=\frac{1}{\sigma \sqrt{2\pi }}exp\left(\frac{-{\left(\sigma_{m}-\mu \right)}^{2}}{2\sigma^{2}}\right)$	*σ*—average value *µ*—standard deviation
Stress amplitudes $\sigma_{a}$	Weibull $p\left(\sigma_{a}\right)=\left(\frac{k}{\lambda }\right){\left(\frac{\sigma_{a}}{\lambda }\right)}^{\left(k-1\right)}e^{-{\left(\sigma_{a}∕\lambda \right)}^{k}}$	*λ*—scale parameter *k*—shape parameter

**Table 13:** Values of the failure accumulation parameter *D*.

Year	Cable LW-20	Cable LW-16	Cable LW-01
2011	0.00	0.00	0.00
2012	0.00	0.00	0.00
2013	1.71 × 10^−6^	7.57 × 10^−7^	1.74 × 10^−9^
2014	3.48 × 10^−6^	1.46 × 10^−6^	3.67 × 10^−9^
2015	5.62 × 10^−6^	1.97 × 10^−6^	4.73 × 10^−9^
2016	8.04 × 10^−6^	2.58 × 10^−6^	5.88 × 10^−9^
2017	1.08 × 10^−5^	3.33 × 10^−6^	7.32 × 10^−9^
2018	1.43 × 10^−5^	4.15 × 10^−6^	8.85 × 10^−9^
2025 *	8.75 × 10^−5^	2.15 × 10^−5^	4.24 × 10^−8^
2050 *	5.02 × 10^−3^	5.80 × 10^−4^	1.26 × 10^−6^
2075 *	3.89 × 10^−2^	2.83 × 10^−3^	6.30 × 10^−6^

* for simulated data.

## Data Availability

The data presented in this study are available on request from the corresponding authors.
